# A novel cancer immunotherapy based on the combination of a synthetic carbohydrate-pulsed dendritic cell vaccine and glycoengineered cancer cells

**DOI:** 10.18632/oncotarget.2908

**Published:** 2015-03-04

**Authors:** Lei Qiu, Jie Li, Shichong Yu, Qianli Wang, Yinghua Li, Zhenlin Hu, Qiuye Wu, Zhongwu Guo, Junping Zhang

**Affiliations:** ^1^ College of Pharmacy, Second Military Medical University, Shanghai 200433, China; ^2^ Department of Chemistry, Wayne State University, Detroit, Michigan 48202, United States

**Keywords:** Cancer immunotherapy, Tumor-associated carbohydrate antigen, GM3 antigen, Cell metabolic glycoengineering

## Abstract

Immune tolerance to tumor-associated carbohydrate antigens (TACAs) has severely restricted the usefulness of most TACAs. To overcome this problem, we selected a sialylated trisaccharide TACA, GM3, as a target antigen, and tested a new immunotherapeutic strategy by combining metabolic bioengineering with dendritic cell (DC) vaccination. We engineered cancer cells to express an artificial structure, *N*-phenylacetyl-D-neuraminic acid, in place of the natural *N*-acetyl-D-neuraminic acid of GM3 by using *N*-phenylacetyl-D-mannosamine (ManNPhAc) as a biosynthetic precursor. Next, we selectively targeted the bioengineered cancer cells by vaccination with DCs pulsed with the GM3 *N*-phenylacetyl derivative. Vaccination with GM3NPhAc-KLH-loaded DCs elicited robust GM3NPhAc-specific T cell-dependent immunity. The results showed that this strategy could significantly inhibit FBL3 tumor growth and prolong the survival of tumor-bearing mice; B16F10 lung metastases could also be reduced. These findings lay out a new strategy for overcoming immune tolerance to TACAs, such as GM3, for the development of effective tumor immunotherapies.

## INTRODUCTION

Immunotherapy is an attractive approach for the treatment of cancer, especially for patients with tumor metastases [1]. One of the most promising approaches to cancer immunotherapy is the use of antigen presenting cells (APC), such as dendritic cells (DC), loaded with tumor-associated antigens (TAAs) because of the potentially superior efficacy and specificity of this approach [2, 3]. However, a major obstacle in the field is the development of immune tolerance to TAAs, which results in the failure of natural TAAs or vaccines formulations with natural TAAs to induce sufficiently effective immune responses. Traditional approaches to improve the immunogenicity of natural TAAs, such as the conjugation of TAAs with large carrier molecules or the coadministration of potent immunological adjuvants, have achieved limited success. Even when vaccines made of TAAs provoke immune responses, they usually only elicit B cell-mediated immunity, rather than the more desirable antitumor T cell immune responses [4–6]. Consequently, the development of a method to overcome the problem of immune tolerance to TAAs will be critical for the success of new cancer vaccines or immunotherapies.

GM3, a sialylated trisaccharide tumor-associated carbohydrate antigen (TACA), is overexpressed by many types of tumors, such as melanoma, leukemia, and pulmonary cancer [7–12]. Recently, we synthesized several GM3 derivatives and demonstrated that unnatural GM3 derivatives, in particular *N*-phenylacetyl GM3 (GM3NPhAc), were more immunogenic than native GM3 and could evoke robust antigen-specific T cell-dependent immunity [13, 14]. For cancer immunotherapy, we exploited a new strategy for selectively targeting the immune response towards cancer cells based on bioengineering. Specifically, we engineered cancer cells to express GM3NPhAc in place of the natural GM3 on the cancer cell surface by using *N*-phenylacetyl-D-mannosamine (ManNPhAc) as a biosynthetic precursor for GM3NPhAc. We have shown that several murine and human tumor cell lines can be metabolically glycoengineered to express GM3NPhAc *in vitro* [15, 16]. Furthermore, *in vivo* ManNPhAc treatment results in abundant GM3NPhAc expression on tumor tissues, but not on normal tissues in tumor-bearing mice [17]. More importantly, bioengineered cancer cells can be selectively targeted by specific immune reactions evoked by conjugate vaccines containing GM3NPhAc [15–17].

In this study, DCs from murine bone marrow loaded with a conjugate of GM3NPhAc and keyhole limpet hemocyanin (GM3NPhAc-KLH) were tested for therapeutic efficacy against cancer in combination with ManNPhAc administration. DCs loaded with GM3NPhAc-KLH could induce robust antigen-specific T cell-dependent immunity. The GM3NPhAc-specific antisera could mediate high cytotoxicity to ManNPhAc-treated B16F10 and FBL3 cells. Lymphocytes isolated from the spleen also showed specific cytotoxicity to the glycoengineered tumor cells. More importantly, the immunity induced by GM3NPhAc-KLH-loaded DCs in combination with ManNPhAc treatment could significantly inhibit tumor growth and metastasis, and also prolong the survival of tumor-bearing mice.

## RESULTS

### Costimulatory molecule expression and IL-12 production by GM3NPhAc-KLH-pulsed DCs

**Figure 1:**
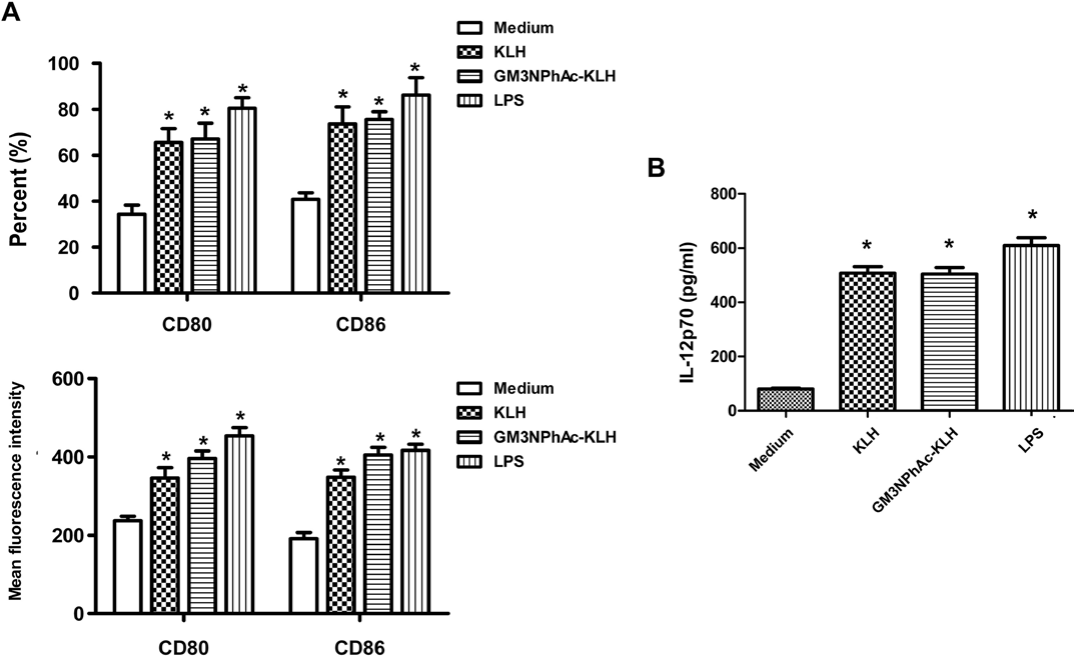
Costimulatory molecule expression and IL-12 production by murine bone marrow-derived DCs DCs were cultured for 24 h with 20 μg/mL KLH, 20 μg/mL GM3NPhAc-KLH, 500 ng/mL LPS, or remained untreated (medium alone). **(A)** Cells were analyzed by flow cytometry. **(B)** IL-12p70 secreted in supernatants was measured by standard ELISA; *n* = 3; *P < 0.05 *vs* untreated controls (medium alone).

Day 5 mouse bone marrow-derived DCs displayed typical morphological characteristics. The DCs pulsed for 24 h with GM3NPhAc-KLH or KLH expressed higher levels of the costimulatory molecules CD80 and CD86, and secreted higher amounts of IL-12p70 compared with unpulsed DCs. LPS activated-DCs were used as a positive control (Figure 1A and 1B).

### The induction of anti-GM3NPhAc antibodies by GM3NPhAc-KLH-pulsed DCs

**Figure 2:**
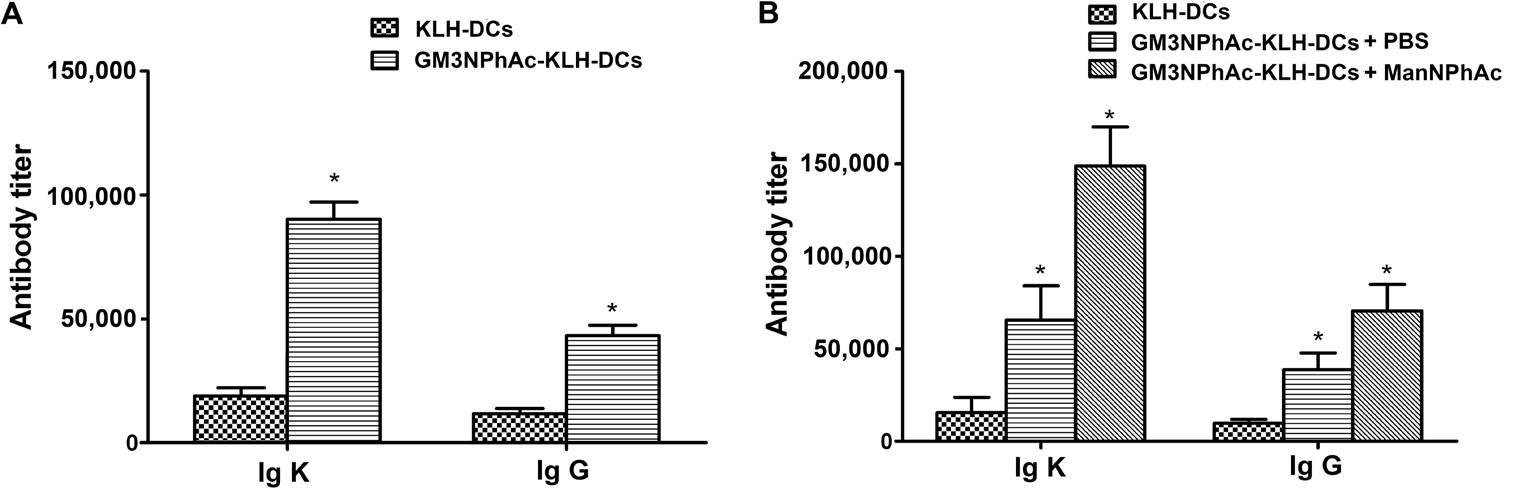
GM3NPhAc-specific total antibody (Igκ) and IgG levels in the sera of immunized mice **(A)** C57BL/6 mice were immunized three times at weekly intervals by s.c. injection of 1 × 10^6^ GM3NPhAc-KLH-DCs or KLH-DCs. Sera were prepared on the day following the final boost. **(B)** Mice were immunized as in (A) One week after the third immunization, each mouse was injected i.v. with 5 × 10^5^ B16F10 cells, followed by daily i.p. injections of ManNPhAc (50 mg/kg/day) for 7 days. Sera were prepared at the end of experiments, i.e. on day 42. Antibodies were assayed by ELISA as described in the Materials and Methods section. For each data set, the mean ± SD for 10 mice per group is shown.

To evaluate the ability of GM3NPhAc-KLH-pulsed DCs to induce GM3NPhAc-specific antibodies, ELISA was used to measure antibody levels in the sera of vaccinated mice. Levels of GM3NPhAc-specific total antibodies (Igκ) and IgG in sera of mice immunized with GM3NPhAc-KLH-DCs increased significantly (*P* < 0.05) compared with those in KLH-DC-vaccinated mice (Figure 2A). Similar results were also observed in B16F10-bearing mice. Interestingly, ManNPhAc treatment, which was used to metabolically glycoengineer cancer cells to express GM3NPhAc, could further increase levels of GM3NPhAc-specific total antibodies and IgG (Figure 2B).

### ADCC and CDC by immune sera towards metabolically glycoengineered cancer cells

**Figure 3:**
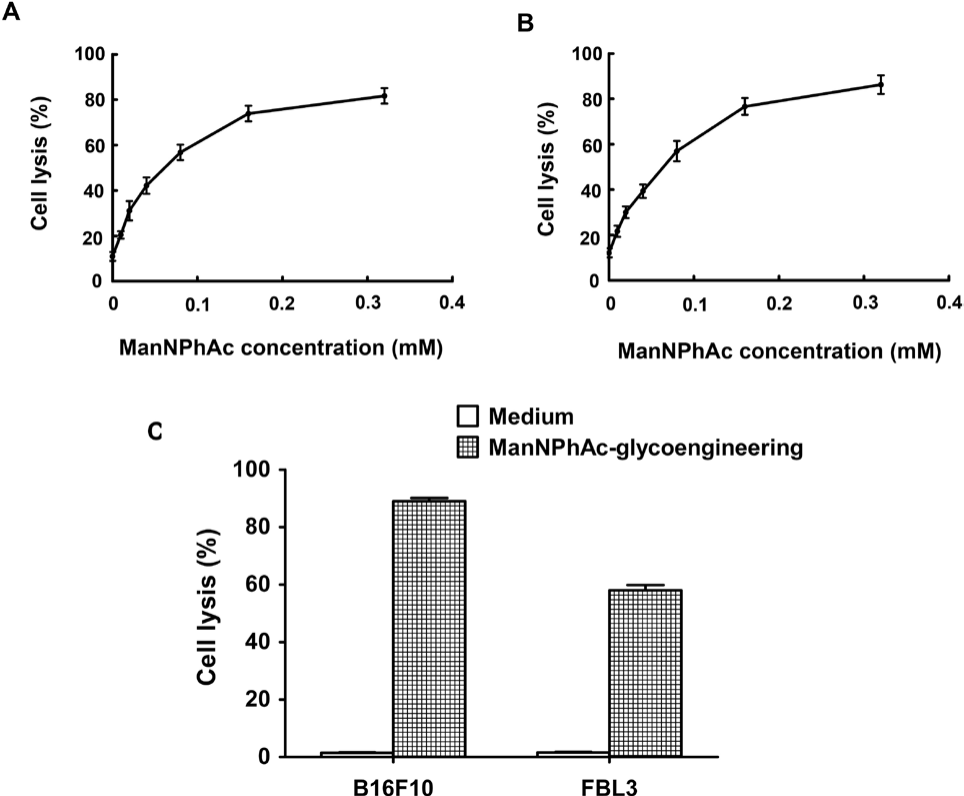
Sera from GM3NPhAc-KLH-DC-vaccinated mice mediated the killing of glycoengineered cancer cells by ADCC or CDC **(A, B)** FBL3 cells were incubated for 72 h with 0, 0.01, 0.02, 0.04, 0.08, 0.16, or 0.32 mM of ManNPhAc. ADCC assays (A) were performed using peritoneal macrophages from healthy mice as effectors with an effector-to-target cell ratio of 100:1. For antibody-mediated CDC assays (B), rabbit complement was used as an effector. **(C)** Anti-GM3NPhAc antisera did not mediate the killing of B16F10 or FBL3 cells by CDC. Cell lysis was evaluated by LDH assays. Each data set indicates the mean ± SD of experiments performed in triplicate.

To assess whether the GM3NPhAc-KLH-DC-vaccinated sera could mediate the killing of glycoengineered cancer cells, B16F10 and FBL3 cells were incubated with various concentrations of ManNPhAc for 72 h. ADCC and antibody-mediated CDC were then measured *in vitro*. Our findings clearly demonstrated that the anti-GM3NPhAc antisera mediated significant cytotoxicity towards ManNPhAc-treated FBL3 cells using either mouse peritoneal macrophages (Figure 3A) or rabbit complement (Figure 3B) as effectors. Similar results were obtained using ManNPhAc-treated B16F10 cells (data not shown). However, anti-GM3NPhAc antisera could not mediate the killing of B16F10 or FBL3 cells (Figure 3C).

### Induction of GM3NPhAc-specific CTL responses by GM3NPhAc-KLH-pulsed DCs

**Figure 4:**
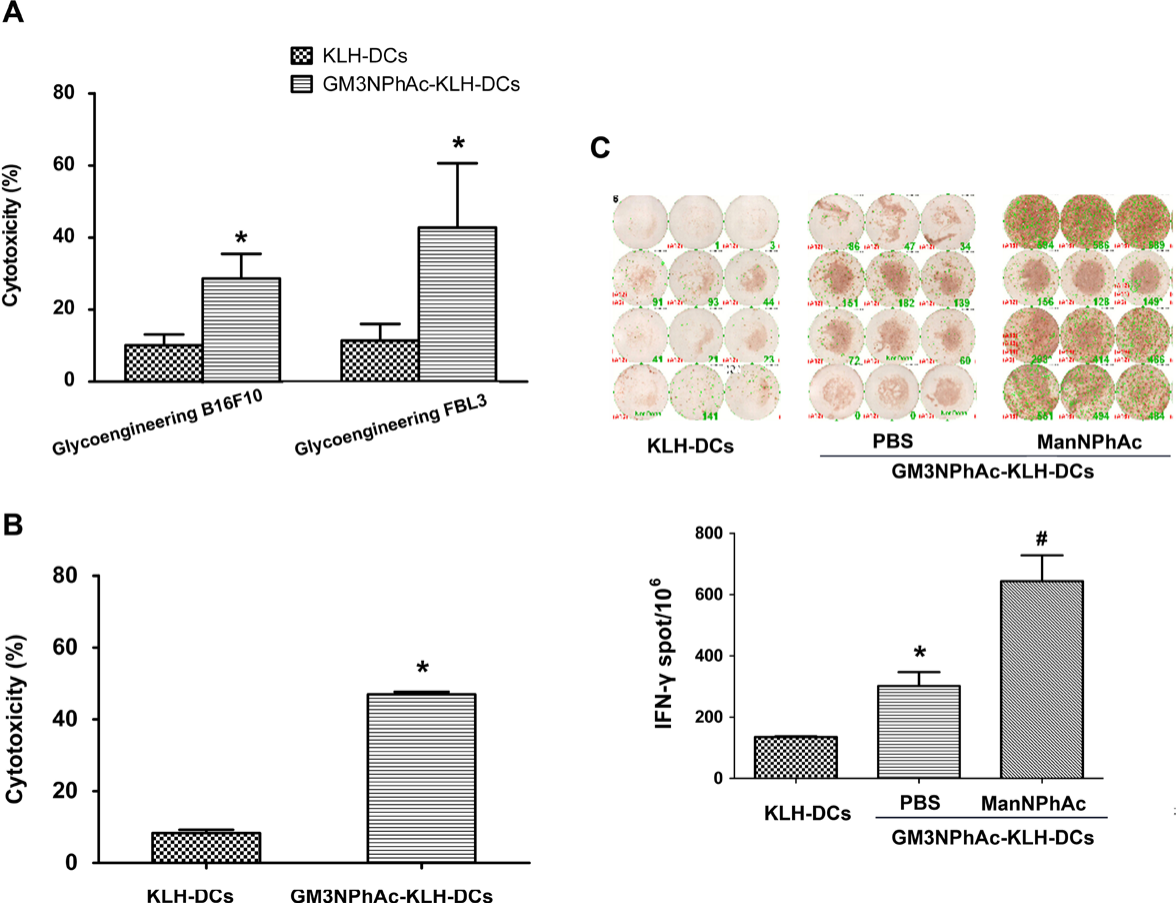
GM3NPhAc-KLH-DCs induce CTL responses **(A)** Mice were immunized as in Figure 2A. Splenocytes from GM3NPhAc-KLH-DC- or KLH-DC-immunized mice were restimulated for 24 h with the corresponding antigens. CTL activity of splenocytes towards glycoengineered B16F10 or FBL3 cells was analyzed by LDH assays at an effector-to-target cell ratio of 100:1; *n* = 10; *P < 0.05 *vs* the KLH-DC group. **(B)** Splenocytes from mice treated as in Figure 2B were tested for CTL activity; *n* = 10; *P < 0.05 *vs* the KLH-DC group. **(C)** ELISPOT assays to determine the number of IFN-γ-secreting lymphocytes from mice treated as in Figure 2B; *n* = 10; *P < 0.05 *vs* the KLH-DC group; # *P* < 0.05 *vs* the GM3NPhAc-KLH-DC and ManNPhAc group. The figure shows results that are representative of four mice.

To evaluate whether DCs pulsed with GM3NPhAc-KLH were capable of inducing CTL responses, GM3NPhAc-KLH-DCs were injected into naive mice. Then, splenocytes from immunized mice were harvested and restimulated for 24 h with GM3NPhAc-KLH for CTL assays. Splenocytes from GM3NPhAc-KLH-DC-immunized mice displayed significant *in vitro* cytotoxicity towards metabolically glycoengineered B16F10 and FBL3 cells (Figure 4A).

To analyze GM3NPhAc-KLH-DC-induced CTL activity in tumor-bearing mice, splenocytes were harvested from B16F10 tumor-bearing mice treated with GM3NPhAc-KLH-DCs and ManNPhAc and were directly used in CTL assays. ManNPhAc-treated B16F10 cells were selectively targeted and killed by these splenocytes (Figure 4B). IFN-γ ELISPOT assays showed that GM3NPhAc-KLH-DC immunizations could markedly increase the number of IFN-γ-producing splenocytes. ManNPhAc treatment could further increase the number of IFN-γ-producing splenocytes (Figure 4C).

### Induction of immune-mediated protection in mice vaccinated with combined GM3NPhAc-KLH-DCs and ManNPhAc treatment

**Figure 5:**
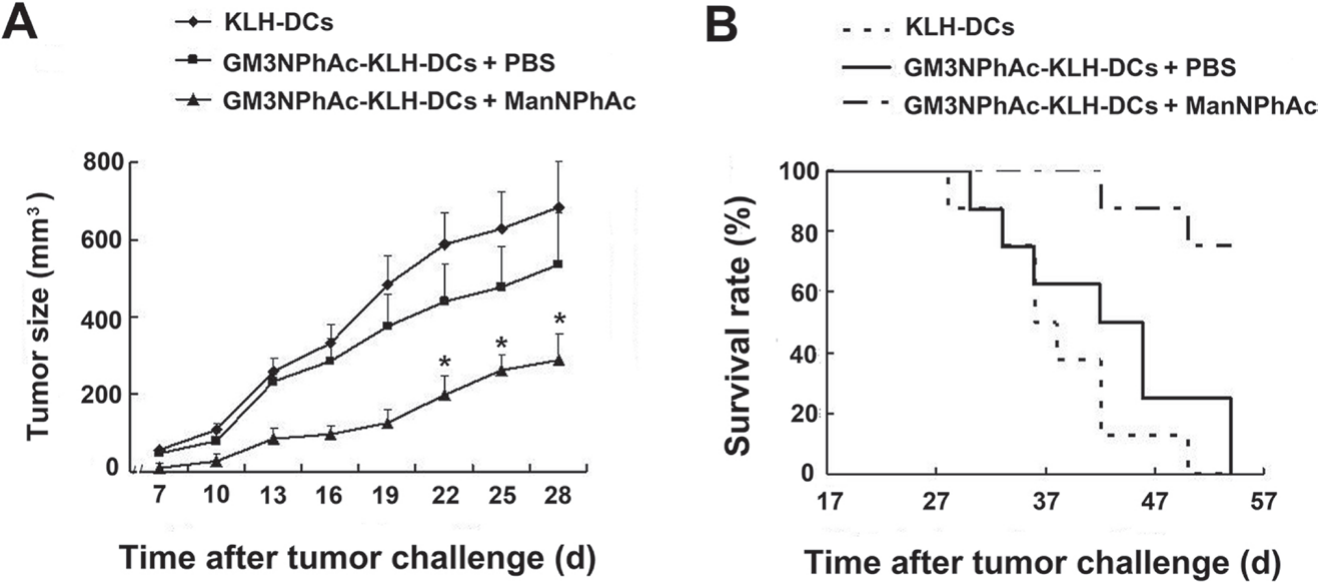
Evaluation of a novel immunotherapy to treat FBL3 cancer **(A)** Tumor sizes and growth rates in mice treated with KLH-DCs, GM3NPhAc-KLH-DCs and PBS, or GM3NPhAc-KLH-DCs and ManNPhAc; *P < 0.05, as compared with the other two treatment groups. **(B)** The survival of tumor-bearing mice treated with KLH-DCs, GM3NPhAc-KLH-DCs and PBS, or GM3NPhAc-KLH-DCs and ManNPhAc.

To test the immune-mediated protection generated by GM3NPhAc-KLH-DC vaccination *in vivo*, we injected mice three times with GM3NPhAc-KLH-DCs at weekly intervals. Mice were inoculated with FBL3 cells by s.c. injection, followed by daily i.p. injections of ManNPhAc (50 mg/kg/day) for 7 days. Mice received GM3NPhAc-KLH-DCs in PBS, or control mice received KLH-DCs. Tumor size was measured using a caliper ruler. Tumors in both groups of control mice grew progressively and developed into palpable tumors (about 1 mm in diameter) 10 days earlier than those in the group treated with GM3NPhAc-KLH-DCs and ManNPhAc (Figure 5A). The tumor size in the treatment group was significantly smaller than that in both control groups (*P* < 0.05). Moreover, GM3NPhAc-KLH-DCs and ManNPhAc treatments could significantly prolong the survival time (*P* < 0.05) of tumor-bearing mice (Figure 5B). All of the mice in the control groups died by day 54. However, 75% of the mice in the treatment group were alive at that time point. The average survival time was 42.6 days for the GM3NPhAc-KLH-DCs and PBS group, and >61.0 days for the GM3NPhAc-KLH-DCs and ManNPhAc group when the experiment was ended on day 66.

**Figure 6:**
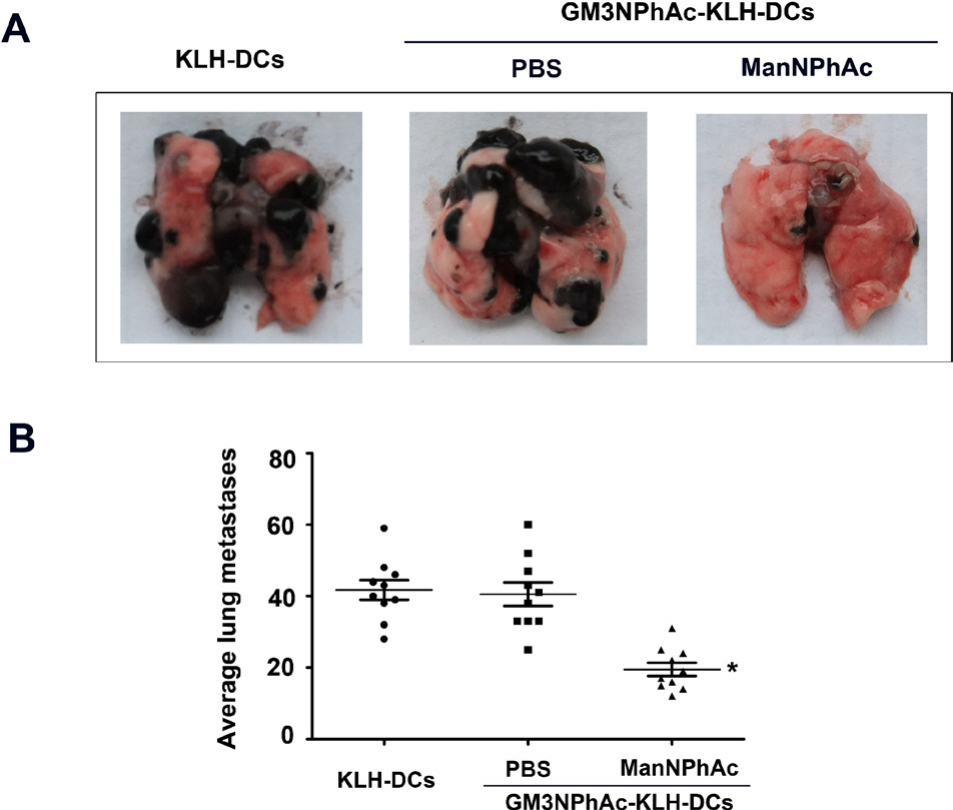
Evaluation of the novel immunotherapy in the B16F10 lung metastasis model **(A)** Representative images of lungs 42 days after the initial immunization. **(B)** B16F10 nodules on the lungs were counted; *n* = 10; *P < 0.05 *vs* the GM3NPhAc-KLH-DCs and PBS group.

We further used the B16F10 lung metastasis model to evaluate the efficacy of the new immunotherapy regimen. In this experiment, mice were treated as described above except that they were also challenged with intravenously (i.v.) injected B16F10 cells. The number of lung nodules was counted 42 days after the initial immunization. Vaccination with GM3NPhAc-KLH-DCs combined with cells metabolically glycoengineered with ManNPhAc led to a dramatic reduction in the number of lung metastases (19.38 ± 7.33 per mouse), which was less than that in both control groups (39.38 ± 13.70 per mouse in the GM3NPhAc-KLH-DCs and PBS group, and 41.89 ± 11.47 per mouse in the KLH-DCs group; Figure 6A and 6B).

## DISCUSSION

TACAs are both the most exposed and often the most abundant TAAs on the surface of cancer cells. Expression of TACAs is closely correlated with tumor progression and metastasis [18, 19]. Therefore, they are ideal molecular targets for immune recognition in cancer immunotherapy [7, 20]. However, a major obstacle in the therapeutic application of TACAs is immune tolerance. This problem has severely restricted the usefulness of most TACAs. We previously demonstrated that some unnatural TACA derivatives could evoke robust T cell-mediated immune responses [13–17, 21–24]. Additionally, cell glycoengineering has been established as a useful technique for the modification of carbohydrate antigens on tumor cell surfaces *in vitro* and *in vivo* [15–17]. These findings render unnatural TACAs derivatives potentially useful for cancer immunotherapy based on the combined application of synthetic vaccines made of unnatural TACA derivatives and cancer cell metabolic glycoengineering. In this study, we exploited a new method to use *ex vivo* conditioned DCs as cellular adjuvants for cancer immunotherapy in combination with cell glycoengineering to enhance immunological recognition and generate targeted immune responses.

DCs are uniquely potent in their ability to capture and process antigens, and they express high levels of MHC-peptide complexes and costimulatory molecules that allow for efficient activation of T cells that can play critical roles in cancer immunotherapy [3, 25, 26]. Our previous studies have shown that DCs generated from peripheral blood mononuclear cells of multiple myeloma patients and loaded with glycoengineered multiple myeloma antigens evoked strong allogeneic stimulatory activity in mixed lymphocyte reactions and efficiently activated CD4^+^ and CD8^+^ T cells. Importantly, these DC-activated T cells were specifically cytotoxic towards the glycoengineered myeloma cells [27]. In our study presented here, we demonstrated that murine bone marrow-DC pulsed with GM3NPhAc-KLH induced a protective immune response against tumor cells expressing GM3NPhAc antigen. Mice vaccinated with GM3NPhAc-KLH-DCs produced high levels of GM3NPhAc-specific total and IgG antibodies. These results are in accordance with previous studies that showed that GM3NPhAc-KLH immunization induced IgG1, IgG2a, and IgG3 antibodies [17], indicating that a helper T cell response had been primed. The development of GM3NPhAc-specific antibodies in mice that were immunized with GM3NPhAc-KLH-DCs correlated with the levels of ADCC and CDC activities. Thus, antibody-mediated cytotoxicity against tumors may be an important mechanism for tumor protection. CTLs are critical effectors in antitumor immunity. To investigate the *in vivo* generation of a GM3NPhAc-specific T cell responses elicited by this DC vaccine, antigen-specific CTL responses were observed *in vitro*. Effector cells obtained from mice immunized with GM3NPhAc-KLH-pulsed DCs could efficiently lyse GM3NPhAc-expressing murine B16F10 and FBL3 cells. Furthermore, GM3NPhAc-KLH-DC vaccination effectively generated IFN-γ-secreting splenocytes, as indicated by ELISPOT assays. Ultimately, we have demonstrated that GM3NPhAc-KLH-DC immunization in combination with ManNPhAc treatment showed robust effectiveness *in vivo* and resulted in the elimination of B16F10 and FBL3 tumors. This novel immunotherapeutic strategy could significantly inhibit FBL3 tumor growth and prolong the survival of tumor-bearing mice. Furthermore, it could prevent B16F10 lung metastases. The results indicate that the use of DC as direct APCs could improve the potency of GM3NPhAc-KLH as a vaccine, which can include both strong T cell-mediated humoral and cellular anti-GM3NPhAc immunity for effective cancer immunotherapy.

Biochemical engineering of TACAs on cancer cell surfaces is the basis for selectively targeted immunotherapy using vaccines made of unnatural TACA derivatives. The biosynthesis of carbohydrates uses no rigid template and is controlled by a series of specific carbohydrate biosynthetic enzymes, including sialyltransferase, which is highly expressed in cancer cells [18, 28]. The flexible substrate permissibility of the enzymes makes it convenient to engineer cancer cells that express GM3NPhAc on the cell surface by using ManNPhAc instead of the naturally occurring N-acetyl-D-mannosamine (ManNAc) molecule as a biosynthetic precursor. Therefore, this bioengineering strategy for immunotherapy could be generally applicable to different TACAs and tumors that express sialo-TACAs. This concept is supported by our previous finding that ManNPhAc, the physiological precursor of N-acetyl sialic acid, could be successfully used *in vitro* and *in vivo* to glycoengineer numerous tumor cells, but not normal cells, to express GM3NPhAc in place of native GM3. It is theoretically possible that the artificial neoantigen could be expressed on normal cells and cause autoimmune reactions. However, we did not observe GM3NPhAc expression on normal tissues, as shown by immunostaining in tumor-bearing mice [17]. Moreover, no remarkable ManNPhAc treatment-related toxicities that affected the weight or general behavior of the immunized mice were observed in these experiments, perhaps because the concentrations of the modified glycans on normal cells are too low to provoke strong immune responses. The limited negative consequences of ManNPhAc usage in mice are thus important for potential human trials in the future.

In summary, we have demonstrated that GM3NPhAc-KLH-DCs can stimulate robust T cell-mediated immunity. This vaccine could significantly inhibit tumor growth and metastasis, and also prolong the survival of cancer-bearing animals when used in combination with ManNPhAc treatment. This work lays out a new strategy to overcome the problem of immune tolerance for the development of effective TACA vaccines. This study also supports the potential benefits of developing new cancer immunotherapies that combine DC vaccination against GM3NPhAc with ManNPhAc treatment for application in humans. We propose that this type of immunotherapy may be applicable to melanoma, leukemia, breast carcinoma, pulmonary cancer, prostatic carcinoma, and other types of tumors that express GM3. Finally, we chose GM3 as a target antigen to confirm the feasibility of this novel immunotherapeutic strategy. It is worth mentioning that this strategy is theoretically applicable to additional sialo-TACAs expressed by other tumor cells.

## MATERIALS AND METHODS

### Reagents

GM3NPhAc-KLH and GM3NPhAc-HSA conjugates, as well as ManNPhAc were prepared according to previously reported methods [13, 15]. The homogeneity and purity of gangliosides was >95%, as determined by TLC and densitometry [22]. Lipopolysaccharide (LPS; Escherichia coli, O26:B6) and KLH were obtained from Sigma (St Louis, MO, USA). Recombinant mouse GM-CSF and IL-4 were purchased from R&D Systems (Minneapolis, MN, USA). FITC- or PE-labeled antibodies (Abs) against murine CD80 and CD86, and isotype control Abs were from BD Pharmingen (San Diego, CA, USA). The alkaline phosphatase-linked goat anti-mouse IgG antibody and Igκ antibody were purchased from Southern Biotechnology (Birmingham, AL, USA). The LDH Assay Kit was purchased from Takara Bio Inc. (Otsu, Japan).

### Animals and cell culture

Six- to eight-week-old female C57BL/6 mice were purchased from Shanghai SLAC Laboratory Animal Co. Ltd. (Shanghai, China). All procedures involving animal treatment and care in this study were approved by the animal care committee of the Second Military Medical University in accordance with institutional and Chinese government guidelines for animal experiments.

A murine melanoma cell line, B16F10, and a murine leukemia cell line, FBL3, were obtained from ATCC (Manassas, VA, USA). Cells were cultured in RPMI-1640 supplemented with 10% heat-inactivated FBS, 100 U/mL penicillin, and 100 μg/mL streptomycin at 37°C in a 5% CO_2_ atmosphere.

### Generation and culture of bone marrow-derived DCs

Murine DCs were prepared and analyzed as previously described [29] with some modifications. Briefly, bone marrow was flushed from the femur and tibia of mice and red blood cells were lysed with 0.84% ammonium chloride. Cells were cultured in RPMI-1640 complete medium for 2 h to allow for adherence. Non-adherent cells were collected and incubated with culture medium supplemented with recombinant murine GM-CSF (10 ng/mL) and recombinant murine IL-4 (10 ng/mL). On day 5, non-adherent cells were harvested as DCs and used for the subsequent experiments.

### DC pulsed with antigen and phenotypic analysis

We incubated 2 × 10^6^ DCs for 24 h with 20 μg/mL GM3NPhAc-KLH, 20 μg/mL KLH, or medium alone. The resulting DCs were referred to as GM3NPhAc-KLH-DCs, KLH-DCs, or medium-DCs (unpulsed). DCs were stimulated with 500 ng/mL LPS as a positive control. Expression of the costimulatory markers CD80 and CD86 was quantitated by flow cytometry using FITC- or PE-labeled Abs against murine CD80 and CD86, respectively. IL-12p70 production was measured in supernatants using a murine IL-12p70 ELISA kit (R&D Systems).

### Preparation of ManNPhAc-glycoengineered tumor cells

FBL3 or B16F10 cells (5 × 10^5^) were cultured in RPMI-1640 complete medium containing different concentrations of ManNPhAc in 6-well plates according to an established protocol [17]. After 72 h incubation, cells were washed twice with PBS and used in subsequent experiments. Unless otherwise indicated, the glycoengineering experiments described in this study were performed using ManNPhAc (1 mmol/L).

### Immunizations

C57BL/6 mice were vaccinated by three subcutaneous (s.c.) injections of 1 × 10^6^ GM3NPhAc-KLH-DCs or KLH-DCs in 0.1 mL of PBS in the lower right flank at weekly intervals. All mice were bled and spleens were harvested on the day following the final boost. Sera were prepared for antibody assays. Splenocytes were restimulated with 10 μg/mL GM3NPhAc-KLH or KLH for 24 h and these splenocytes were used as effector cells for cytotoxic T lymphocyte (CTL) assays.

### Antibody assays

Specific anti-GM3NPhAc antibody was measured by ELISA as previously described [15, 17, 24]. Briefly, GM3NPhAc-HSA was coated on ELISA plates. Serial dilutions of sera from immunized mice were then added and incubated at 37°C for 2 h. After washing, plates were incubated with 1:1000 dilutions of alkaline phosphatase-linked goat anti-mouse κ, or IgG antibody for 1 h at room temperature. Finally, plates were developed with PNPP substrate. The absorption (A) value was measured using a plate reader at a 405 nm wavelength. To measure titers, OD values were plotted against dilution numbers, and a best-fit line was obtained. The slope of this line was used to calculate the dilution number at which an OD value of 0.5 was achieved, and this dilution number indicated the antibody titer.

### Antibody-dependent cell-mediated cytotoxicity (ADCC) and antibody-mediated complement-dependent cytotoxicity (CDC) assays

ADCC and antibody-mediated complement-dependent cytotoxicity (CDC) activities were determined as previously described [15, 17] using a LDH cytotoxicity detection kit. Briefly, ManNPhAc-glycoengineered FBL3 or B16F10 tumor cells (1.5 × 10^4^ cells/well) were used as target cells and incubated with antisera derived from GM3NPhAc-KLH-DCs immunized mice (1:20 dilution in DMEM) at 37°C for 2 h. Thereafter, peritoneal macrophages isolated from healthy mice or rabbit complement serum (diluted 1:10 in DMEM) were added to each well as effectors. Plates were incubated at 37°C for another 18 h for ADCC or 1 h for CDC assays, and then cell supernatants were harvested to detect cell lysis in a LDH assay using a LDH cytotoxicity detection kit according to the manufacturer's instructions. Results are expressed as the percentage of cell lysis.

### CTL assays

CTL assays were performed as previously described. [17] Briefly, splenocytes from immunized mice were cultured for 24 h with or without 2 μg/mL of the corresponding antigen, KLH or GM3NPhAc-KLH. These splenocytes were used as effector cells and were co-cultured with FBL3 cells or ManNPhAc-glycoengineered FBL3 cells (target cells) at an effector-to-target cell ratio of 100:1 in RPMI-1640 with 10% FBS for 24 h. Cell-free supernatants were collected and analyzed using the LDH assay.

### IFN-γ enzyme-linked immunosorbent spot (ELISPOT) assay

IFN-γ-producing splenocytes were quantified by ELISPOT assay as previously described [17]. In brief, 1 × 10^6^ splenocytes were added to IFN-γ ImmunoSpot plates (Dakewe, Shenzhen, China) and incubated at 37°C for 18 h. After washing, plates were incubated with biotin-conjugated anti-IFN-γ mAb, followed by the addition of streptavidin-alkaline phosphatase. Finally, the activator solution (supplied with the ImmunoSpot kit, Dakewe) was added to each well for spot development. The spots were counted using a microplate reader.

### Immune-mediated protection generated by the GM3NPhAc-KLH-DCs vaccine combined with ManNPhAc treatment

To evaluate the efficacy of the GM3NPhAc-KLH-DCs vaccine to inhibit tumor growth or prolong the survival of tumor-bearing animals, C57BL/6 mice were vaccinated by three s.c. injections of 1 × 10^6^ GM3NPhAc-KLH-DCs in 0.1 mL of PBS administered at weekly intervals. One week after the third immunization, each mouse was inoculated with 5 × 10^5^ FBL3 cells s.c. into the left flank, followed by daily i.p. injections of ManNPhAc (50 mg/kg/day in 0.1 mL of PBS) for 7 days. Mice received immunizations with GM3NPhAc-KLH-DCs plus PBS; mice immunized with KLH-DCs alone were used as a control group. Tumors that developed in immunized mice were monitored and measured using a caliper ruler. Tumor size was calculated using the formula 0.4 × (A^2^ × B), (where B represents the largest diameter and A indicates the diameter perpendicular to B). To assess the impact of the immunotherapy on the survival of animals, mice were treated as described above and kept under close observation. Animals were maintained until they died of cancer or, if a tumor reached the size of 700 mm^3^, the animals were euthanized and similarly scored as ‘died of cancer.’

To evaluate the efficacy of the GM3NPhAc-KLH-DCs vaccine to inhibit tumor metastasis, 5 × 10^5^ B16F10 cells were injected into the tail vein of each mouse. GM3NPhAc-KLH-DC immunization and ManNPhAc treatment were carried out using the same protocols as described above. On day 42, all mice were sacrificed. The nodules in the lungs were counted and splenocytes were isolated for both the CTL assay and the enzyme-linked immunosorbent spot (ELISPOT) assay for IFN-γ-secreting lymphocytes.

### Statistical analyses

All experiments were carried out in triplicate, and all data are presented as the mean ± SD. Statistical analyses were performed using the Student's *t*-test or two-way ANOVA. Differences were considered to be statistically significant when *P* < 0.05. All statistical analyses were performed using GraphPad Prism 5.0 software (La Jolla, CA, USA).

## Abbreviations

TACAs: Tumor-associated carbohydrate antigens
KLH: Keyhole limpet hemoeyanin
HSA: Human serum albumin
CTL: Cytotoxic T lymphocyte
