# Erratum: A novel cancer immunotherapy based on the combination of a synthetic carbohydrate-pulsed dendritic cell vaccine and glycoengineered cancer cells

**DOI:** 10.18632/oncotarget.6369

**Published:** 2015-11-23

**Authors:** Lei Qiu, Jie Li, Shichong Yu, Qianli Wang, Yinghua Li, Zhenlin Hu, Qiuye Wu, Zhongwu Guo, Junping Zhang

Oncotarget. 2015; 6:5195-5203

***PMID:*** *25760071*

Present:

**FINANCIAL SUPPORT**

This work was supported by the National Natural Science Foundation of China (No.30728032, 21202200).

Correct:

**FINANCIAL SUPPORT**

This work was supported by the National Natural Science Foundation of China (No.30728032, 21202200) and **R01 CA095142/CA/NCI NIH HHS/United States**.

